# Mobile X-ray services in nursing homes as an enabler to healthcare-in-place for residents: informal carers’ views

**DOI:** 10.1186/s12877-023-04130-7

**Published:** 2023-07-25

**Authors:** Joanne Dollard, Jane Edwards, Lalit Yadav, Virginie Gaget, David Tivey, Guy J. Maddern, Renuka Visvanathan

**Affiliations:** 1 Adelaide Geriatrics and Training with Aged Care (GTRAC) Centre, Adelaide Medical School, Faculty of Health and Medical Sciences, University of Adelaide, Woodville South, South Australia Australia; 2 Basil Hetzel Institute for Translational Health Research, Central Adelaide Local Health Network, Woodville South, South Australia Australia; 3 Discipline of Surgery, The Queen Elizabeth Hospital, The University of Adelaide Surgical Specialties, University of Adelaide, Woodville South, South Australia Australia; 4 Royal Australasian College of Surgeons, Adelaide, South Australia Australia; 5 Aged and Extended Care Services, The Queen Elizabeth Hospital, Central Adelaide Local Health Network, Woodville South, South Australia Australia

**Keywords:** Nursing homes, Geriatric assessment, Mobile health units, Diagnostic services, X-Rays, Qualitative research, Informal caregivers

## Abstract

**Background:**

Informal carers (ICs) of residents living in nursing homes (NH) have a key role in the care of residents, including making decisions about and providing care. As radiology has a role in decision making about care, it is important to understand IC’s perspectives about resident’s use of mobile X-ray services (MXS). The aim was to explore the perspectives of ICs of residents living in nursing homes about the use of MXS.

**Methods:**

From November 2020 to February 2021, twenty ICs of residents living in four nursing homes in different areas of one Australian city participated. Their perspectives of MXS, including benefits and barriers, were explored in semi-structured interviews. Data were analysed using thematic analysis.

**Results:**

ICs were resident’s children (80%) and spouses (20%). One resident had received a MXS. Four themes were developed: (1) a priority for resident well-being, where ICs were positive about using MXS, because residents could receive healthcare without transfer; (2) MXS could reduce carer burden; (3) economic considerations, where MXS could reduce health system burden but the MXS call-out fee could result in health inequities; and (4) pathways to translation, including the need to improve consumer awareness of MXS, ensure effective processes to using MXS,, consider nursing home staff levels to manage MXS and ICs expectations about quality and availability of MXS.

**Conclusions:**

ICs consider MXS can benefit resident well-being by potentially reducing transfers to hospital or radiology facilities and advocated equitable access. ICs cautioned that the quality and safety of healthcare delivered in nursing homes should equal what they would receive in hospitals.

**Supplementary Information:**

The online version contains supplementary material available at 10.1186/s12877-023-04130-7.

## Background

Radiology imaging has a key role in the diagnosis and decision-making regarding management of the health of residents. Many residents living in Australian nursing homes (NHs) (known also as aged care homes, residential aged care facilities, long-term care) are currently transferred to a hospital emergency department for radiology imaging [1]. For many, this trip translates into a hospital stay even when the required care could have been provided in the NH. A hospital transfer can be distressing to residents, exposing residents to unfamiliar environments, and taking them away from staff they know and trust [2]. Further, a hospital transfer exposes residents to potential hospital acquired complications, such as delirium, falls and pressure injuries [3, 4]. For these vulnerable and frail residents, access to timely diagnostics in their usual place of residence could lead to healthcare-in-place, instead of transfer to hospital. This, however, requires additional support to ensure safe and high quality healthcare-in-place [5].

The pressures on emergency departments are increasing significantly, in part driven by population ageing, resulting in ambulances parking outside emergency departments (with patients) due to a lack of clinical space in emergency departments (ramping) [6]. As a result, there are increasing calls for models of care to be developed for residents of NHs [6]. In 2019–2020, there were 245,000 permanent residents of NHs across Australia, with the number expected to increase over coming decades [7]. Not surprisingly, there is strong interest in developing and testing new models of care that better support the delivery of healthcare-in-place, in NHs. For example, whilst results are yet to be published, a stepped-wedge randomised controlled trial has been conducted in 12 Queensland NH. It investigated hospital avoidance interventions, including education and training, tools to support decision-making, use of diagnostic equipment (e.g. bladder scanners) and implementation facilitation, with the primary outcome of hospital bed days [8].

In Australia, a universal public health insurance scheme (Medicare) funds health services outside of public hospitals through Medicare Benefit Schedule listed rebates to non-government healthcare providers. All working Australians pay a Medicare levy as part of their income tax, 2% of their taxable income. To encourage high income earners to take up private insurance to fund private hospital care, an additional Medicare surcharge ranging between 1 and 1.5% is charged to those without private insurance.

Medicare Benefit Schedule rebates have existed for radiology services. The use of plain X-rays for reasons residents commonly present to emergency department (e.g. fall-related injury, pneumonia, heart failure, acute abdomen and bowel obstruction) has been increasing over time. Our research identified a 12% increase in the use of the Medicare Benefit Schedule rebate between 2009 and 2016 [9]. Mobile X-ray services (MXS) have been proposed as a means of reducing emergency department presentation. For example, a 2015 Australian pre post study demonstrated an 11.5% reduction in emergency department presentations for plain X-rays in the year following commencement of the MXS delivered by a hospital [10].

Prior to November 2019, whilst non-government radiology providers were also performing MXS in Australian NHs where these providers claimed Medicare Benefit Schedule rebates for the conduct of the X-rays, they also charged residents a call-out fee to cover the costs of transporting equipment to and from the NH. Recognising that this call-out fee was a barrier to increased and equitable uptake of such services in NHs and appreciating the potential for such services as a hospital avoidance strategy, the Australian Government listed a Medicare Benefit Schedule rebate (item 57,541) to fund call-out fees in November 2019. This rebate was available for specific conditions that could otherwise result in a resident transfer to the emergency department (skeletal X-rays of extremities, shoulder, pelvis, ribs and sternum after falling, chest X-ray for suspected pneumonia and heart failure, plain abdominal X-ray for acute abdomen or suspected bowel obstruction). The rebate was payable where the X-ray was requested by a general practitioner after they had assessed the resident in person [11].

This qualitative research was funded through the Medical Research Future Fund as part of a larger program of mixed-method research [9, 12, 13] to inform the Australian Government Department of Health’s evaluation of the rebate for the call out fee. Our broader aim in using qualitative research was to explore the applicability of MXS with health and aged care stakeholders, residents and informal carers, as this was yet to be clearly established in the literature. We interviewed stakeholders involved in the delivery of NH healthcare and residents. We found that MXS were viewed as valuable for residents, by improving accessibility to radiology diagnostics to support the provision of healthcare-in-place [14]. Residents were positive about MXS as a means of avoiding burden on their informal carers (ICs) who support them during transfers to healthcare facilities for radiology investigations [15].

ICs provide unpaid care to older adults with whom they have a social relationship [16]. In NHs, ICs have a crucial role in ongoing instrumental activities, such as managing finances and transportation to appointments, providing physical, social, practical, and or emotional support, and advocacy [17, 18]. Further, ICs have a crucial role when a residents’ care needs change. ICs can detect changes in a residents’ condition before others [17], and may advocate for care, including investigation. They can be involved in complex decision-making about resident care, such as whether to transfer to hospital, whilst considering care management in the NH [19]. In any case, ICs may also have to give formal consent to radiology investigation for some residents who are unable to consent themselves. Therefore, ICs are key stakeholders in resident well-being in NHs and understanding their perspectives is crucial. It is therefore surprising that to date no research has been published on the perspectives of ICs of NH residents in relation to MXS; a major gap in the literature addressed in this study.

The specific aim of this qualitative study therefore was to explore the perspectives of ICs of residents living in NH about benefits of MXS and barriers to its use in clinical care. The research objectives were to explore with ICs: what they thought was important to residents’ lives; knowledge of MXS; perceived benefits, costs, risks, barriers and facilitators; and their willingness to pay for MXS.

## Methods

The University of Adelaide Human Research Ethics Committee provided ethics approval for the research protocol: H-2020-197. All participants provided written informed consent (with two participants providing consent via email) prior to participation in interviews.

The first author (an experienced qualitative researcher) conducted interviews between 04/11/2020 and 12/02/2021.

### Study design and setting

The researchers approached six aged care organisations to participate but two declined. The participating organisations were offered a $500 honorarium to identify a NH and recruit ICs for this study (as well as recruiting residents and staff for two other related studies) from NHs located across one Australian city. NHs varied in bed size (see Table 1), with between 50 and 100 beds to 151–200 beds, with one NH being more culturally and linguistically diverse.

### Participant recruitment

The aim was to interview approximately twenty participants or until data saturation (defined as few additional insights). Participants needed to meet inclusion criteria which included being: (a) an IC of a resident living in a participating NH; (b) aged ≥ 18 years; and able to: (c) provide informed consent; (d) communicate effectively; and (e) engage in a telephone or videoconferencing interview. Preference was given to recruiting ICs of residents who had experienced a MXS in the previous twelve months.

Staff from the four NH approached eligible ICs, explained the study and provided information sheets and consent forms. Once NH staff obtained informed consent, the interviewer telephoned ICs to reiterate the information about study participation, to answer any questions, to schedule a telephone or videoconference interview at a day and time that suited them,. The interviewer verbally reconfirmed consent with participants prior to commencing the interview.

### Data collection

The semi-structured interview guide was developed de novo for this study, based on literature and research team experience (Supplementary Material 1). The broader research team reviewed the interview guide. The first author conducted two mock interviews via telephone with ICs of residents, recruited through researcher personal networks (data not included). The interview explored what participants considered to be important to residents’ lives and their knowledge about MXS. Following a brief description of what MXS entails, participants were asked about the pros and cons of MXS, their preferences for an X-ray delivered at the ED versus a MXS, and for which health conditions or situations. They were also asked about their willingness to pay for a MXS call-out fee. From the 8th interview, a question was asked about what skills radiographers needed to perform MXS in NH, in response to emerging findings. Issues arising within interviews were also explored.

At the end of each interview, the interviewer summarised the discussion, giving participants the opportunity to correct and add to the summary, and collected demographic information (age, gender and relationship to resident). Field notes were written immediately after the interview. Data saturation was noted at the 17th interview and three more interviews were conducted. Interviews were audio-recorded and transcribed verbatim by a professional transcriber. The first author de-identified transcriptions and checked for accuracy against audio-recordings.

As a quality check, another researcher reviewed two interview transcripts for interview technique. The research team met frequently to discuss recruitment, interviews, and the interview schedule.

After the interviews, NH clinical staff collected resident demographic information (related to participants) from medical records (age, gender, years lived in NH, dementia diagnosis), and reason for X-rays done in the last 12 months and their location (emergency department, community radiology and/or MXS) .

### Data Analysis

Data relating to participant and resident demographics and resident X-rays were entered into SPSS 28 (Armonk, NY: IBM Corp.) and are presented descriptively. Analysis of the interview data was guided by a six-phase process of thematic analysis [20]. An inductive approach was used to generate themes derived from data, as well as being sensitive to the literature. This included the researchers becoming familiar with the dataset (by reading and re-reading transcripts and field notes). The first author, along with two other experienced qualitative researchers, independently coded three transcripts. They discussed and developed initial codes, which the first author used to code remaining transcripts. During coding, the three authors met and reviewed and refined coding. The list of codes was meaningfully grouped and developed into potential themes and sub-themes to answer the research question. Themes and sub-themes were reviewed and defined, and the report written. Two further researchers participated in peer debriefing. NVivo 12 (QSR International Pty Ltd.) was used to assist management of data analysis. Researchers were reflexive about their personal and professional background and assumptions could influence data collection, analysis and interpretation and discussed this within researcher meetings. At the beginning of the study, the first author wrote her own assumptions and experiences that sensitised her to the topic. The authors had no prior relationships with ICs or residents in this study.

## Results

NH staff approached 24 ICs (Table 1); one IC declined and three did not respond (83% response rate). Twenty participants were interviewed, either one-on-one (n = 18) or one-on-two (n = 2) with participants’ partners. Interviews were conducted via telephone (n = 16) or videoconferencing (n = 4) and ranged between 35 and 90 min (median = 47 min, total 1,018 min).

### Participant characteristics and resident characteristics and X-rays

Participants included 13 (65%) daughters, 3 (15%) sons, 3 (15%) wives and 1 (5%) husband of residents. The majority (85%) of residents had lived in the NH for ≥ 2 years and 65% had dementia. In the 12 months before ICs were interviewed, 4 (20%) residents had an emergency department X-ray (2 X-rays fall-related; 1 X-ray post-surgery; 1 X-ray pleural effusion/pulmonary atelectasis), 2 (10%) residents had a community radiology (1 X-ray fall related; 1 X-ray not fall-related) and 1 (5%) resident had a MXS (falls-related).

**Table 1:** Recruitment of informal carers per nursing home and number and location of X-rays for the residents they cared for

NH (bed numbers)	Informal carers recruited per NH N (%)	Informal carers declined/no response N	Residents with X-ray in 12 months pre interview N	X-ray location
NH A (151–200)	5 (25)	0	2	ED, CR
NH B (101–150)	5 (25)	0	1	ED
NH C (101–150)	6 (30)	2 no response	2*	ED x2, CR
NH D (50–100)	4 (20)	1 declined, 1 no response	1	NH

NH: Nursing Home; ED: Emergency Department; CR: Community Radiology; *1 participant received an X-ray in ED and CR

### Themes

Four themes are presented. The first theme, ICs’ priority of maintaining resident well-being could be facilitated by using MXS, included three sub-themes: Resident well-being was a priority to ICs, Perceived benefits of MXS to residents’ well-being and Nursing home equipment to manage MXS for resident benefit. The second theme was reduced carer burden. The third theme, ICs’ economic considerations of using MXS included two sub-themes: Cost benefits to health system and Cost benefits of MXS call-out fee for individuals. The fourth theme, was pathways to translating MXS into NH to meet residents’ and ICs’ needs and expectations and included five sub-themes. They included Awareness of and need for promotion of MXS, Effective processes to using MXS, Nursing home staff levels to manage MXS and care process, Expectations of quality of MXS and Expectations about MXS availability. Exemplar quotes are provided as evidence of the themes (edited to reduce words) (further quotes are provided in Additional File 2).

#### Theme 1: ICs’ priority of maintaining resident well-being could be facilitated by using MXS

ICs perceived that resident well-being was maintained, fostered or optimised through the NH providing an ongoing supportive environment. ICs perceived that if residents could remain in their supportive NH environment, then resident well-being could be maintained, and, therefore, using MXS in the NH could optimise resident well-being. In contrast, ICs perceived that residents having to leave the supportive NH environment could disrupt resident well-being, which ICs wanted to avoid. Further, using equipment that were available in the NH could benefit residents.

*Sub theme 1.1 Resident well-being was a priority to ICs.* Residents’ well-being was a priority for participants, including residents’ ability to remain engaged, socialise, and be in the company of familiar people. Residents remained in an environment where they felt safe, secure and comfortable, whilst being supervised by familiar staff.

That she’s comfortable. That’s she’s safe and that she sees her family members. … The fact that everyone’s looking out for her and her needs are being met **(NH A; ID11)**

ICs of residents with particular vulnerabilities, notably impaired cognition, mobility and ability to communicate, as well as sensory loss, and/or poor prognosis, expressed a strong preference for MXS. Remaining in a familiar environment, with familiar staff and routines was regarded by ICs as very important for such residents.

If they can do it in-house, it would be a lot better. Especially old people with mobility issues and dementia **(NH D; ID19)**

*Sub theme 1.2: Perceived benefits of MXS to residents’ well-being.* ICs gave a very positive evaluation of the concept of the MXS, because of benefits for residents. Participants perceived the benefit of MXS was to residents’ well-being, as residents remained in their familiar environment, with familiar staff and routines. They thought this minimised residents’ emotional distress, as well as avoiding the disruption and burdens of being transferred, which was carers’ preferences. Further, participants recognised residents also received an X-ray that they needed.

I can see it as a very, very good idea. … Because you’re still dealing with very aged people. You’re not there because you’re well. If you’re well and able-bodied, you’re not in a nursing home **(NH A; ID04)**

I see their environment that they live in is like a security blanket. So the less you’ve got to take them away from it, the more settled they become and stay **(NH C; ID27)**

It would be not having to go out, so not distressing to her, but also to be able to get an X-ray (**NH C; ID28**)

Some participants thought that the use of MXS could be extended to older adults living at home and people with disabilities, whose well-being would benefit from receiving an X-ray at home.

I used to work down at [disability services], and I think it would work very well … there was a lot of people down there that would definitely benefit from just staying where they are because they’re in a familiar place with familiar staff **(NH D; ID21)**

The one IC with a relative who received a MXS post fall was transferred to hospital. Whilst the MXS did not result in avoiding transfer, the IC still viewed that the MXS had the *potential* to minimise emotional distress.

Then they were sending her off to hospital … they must have found something wrong but if they didn’t, they wouldn’t have to put her through the trauma of going to hospital (**NH D; ID20**)

*Sub theme 1.3: Using equipment available in the NH to manage MXS for resident benefit.* Some participants thought that there were other benefits for residents of having MXS due to NH’s equipment (up/down beds, lifters), which made the process easier for residents. These were not always available in the radiology department. One participant, however, thought that the NH beds might be too soft to position the resident for an X-ray.

Old people couldn’t hop up on that bed [fixed X-ray] … Whereas in the nursing home, they have the beds that go up and down so that they can be placed or put there on a lifter **(NH B; ID15)**

#### Theme 2: reduced carer burden

Participants described their role of needing to transport, accompany, care and or advocate for residents when they were transferred to have an external X-ray, often at short notice. This experience was taxing for many ICs, particularly managing residents’ reduced mobility and some ICs described being with the resident as stressful. This was disruptive for ICs, but participants undertook this role without complaint, as they saw it was their responsibility to care for their relative.

You are now losing sleep rapidly, because you might be there until four in the morning. You cannot leave him, because he doesn’t have language and … he’s not cognitive. You’re an interpreter and a family carer and a concerned child of a parent who’s got cognitive issues **(NH A; ID05)**

There’s no one else here that can really do … it becomes my responsibility (**NH A; ID09)**

Participants expressed that the use of MXS could lower carer burden, given that ICs would not need to transport or escort residents to emergency department or community radiology. Some ICs were reassured that they did not necessarily need to be with the resident, relying on familiar staff to provide reassurance, while others would still plan to be with the resident to reassure them, as well as ensure clinical information and care flowed.

If … they’ve called them [MXS] in ... For example in the middle of the night for us to get there, because I’ve got nearly an hour’s drive to get to Mum, then it wouldn’t be such a rush for us to be traumatised in the middle of the night to be up, ready, dressed and out of the door and race there if things have already been commenced **(NH A; ID11)**

I think she [resident] would cope. I mean, if it was an emergency and nobody [family] could come, she would cope, because the staff there are fairly reasonable. She knows them … They’ll hold her hand and all that sort of thing **(NH D; ID18)**

#### Theme 3: ICs’ economic considerations of using MXS

ICs discussed economic considerations of using MXS in the NH. Some ICs could see the positive economic impact at the broader level of the health system, by reducing cost to an overstretched acute care public health system. With the direct cost of radiology shifting to the user, ICs weighed up how much and under what conditions they would be prepared to pay for the benefit of MXS.

*Sub-theme 3.1: Cost benefits to health system.* Some participants described health system benefits, such as reducing hospital system costs and ambulance ramping outside emergency departments.

When you start looking at ambulance costs and things like that and also clogging up hospitals and ramping, which we experienced, … there’s actually some very positive aspects from a … general business perspective, as well, from the government’s point of view **(NH C; ID23)**

*Sub-theme 3.2: Cost benefits of call-out fee for MXS to individuals.* The only participant who had previously paid a required upfront call-out fee saw the process of paying upfront, in terms of contacting family to pay, as a potential barrier to others using the MXS.

To think that they want money first, that’s a hole, because somebody’s got to pay it. Mum can’t pay it ... If we were away on a holiday somewhere and couldn’t be contacted and mum needed an X-ray, mobile X-ray would be the last people they could call because they want their money first **(NH D; ID21)**

Participants had varying expectations as to who should cover the call-out fee, with some expecting the government, private insurance or the NH to cover part, or all, of the call-out fee.

There has to be a way that maybe there’s a shared cost, that government pays so much and the nursing home pays like a service fee, like a yearly fee **(NH C; ID27)**

Many participants were willing to pay for the MXS call-out fee, with the highest amount nominated being $500, although they were aware that others, particularly pensioners, might not consider the fee affordable.

I’d probably pay $50 to $100 … I think that would put a lot of people at a disadvantage depending on how much the gap payment was. I think if it was $50 it would probably be more manageable for a lot more people **(NH D; ID19)**

Some participants were willing to pay for some situations (e.g. urgent X-ray) but reluctant to pay for others (e.g. routine X-ray).

If it was necessary for her to have it [MXS], yes, we would pay. … If it wasn’t something that needs to be done in a rush and we could take her to [community X-ray], we would probably go that way, especially knowing there was a fee **(NH C; ID37)**

#### Theme 4: pathways to translating MXS into NH to meet residents’ and ICs’ needs and expectations

ICs identified potential issues and expectations for translating MXS into NH practice, to promote the uptake of MXS and meet the needs and expectations of residents and ICs.

*Sub-theme 4.1: Awareness of and need for promotion of MXS.* There was a low-level awareness of MXS, with many of the participants either not at all aware or only vaguely aware of it. Some participants were aware of MXS as the NH had organised, or had tried to organise, a MXS for residents.

I had no idea that mobile X-rays existed **(NH C; ID24)**

To improve knowledge and facilitate the use of MXS, ICs gave suggestions about how residents could be informed about MXS. This included information on entry to a NH, via NH newsletters, brochures and one-on-one information, as well as brochures provided at NH or general practitioner’s reception rooms. Participants would also like to be given the information and option of MXS at the time residents needed an X-ray.

You can tell us at the beginning, but we’re probably going to forget about that. … When it comes to look, ‘your dad’s had a fall, we think he needs to be X-rayed, would you like us to call the mobile X-ray?‘ That seems to be a fairly simple way of going about it. …I think it should be promoted. … at [NH], they have their pamphlets in there for everything ... What’s wrong with having a pamphlet in there saying about mobile X-rays? **(NH C; ID36)**

*Sub-theme 4.2: Effective processes to using MXS*.

ICs thought that the NH should have training and protocols in place so that MXS can be utilised in an effective way with minimal delays and miscommunication. This includes some participants stating that NH staff should be provided with information and trained about how to access MXS.

You would have to know that the staff there would be able to handle whatever process there is to getting them to … the mobile X-ray **(NH A; ID04)**

It also included improving processes about the upfront payment. The only participant who had previously paid the required upfront fee suggested the NH could have an agreement with families to give permission for the use of MXS and to pay the upfront call-out fee.

Everybody that’s there needs to know that [call-out fee] and agree to say ‘yes or no’. ‘Yes, we’ll pay a $90 fee to have an X-ray come to them’ or ‘we won’t’ and then the home knows which way to make the call **(NH D; ID21)**

It also included that when the MXS was booked, participants wanted to know the scheduled time. ICs stated this would assist their decision-making, as they would not choose a MXS if the resident was going to have to wait longer than was acceptable. They indicated this would also help them to plan their own day, especially where they wanted to be with the resident.

Totally, to give you a bit of an idea of what the wait is, … otherwise, you’re just waiting in a vacuum **(NH B; ID32)**

That wouldn’t be good enough for me. I’d say “right, well I think we’ll take Mum to [community radiology]” **(NH C; ID37)**

*Sub theme 4.3: Nursing home staff levels to manage MXS and care process.* Participants had mixed views about the capacity of NH staffing levels to support the MXS, as well as observe the resident. Some conjectured that the MXS process might take staff away from caring for other residents.

If the staff have to get them onto the bed, I guess it would be more staff time to help. But … that … would be balancing out against the staff time that would take for them to get elsewhere anyway … It’s always a staffing thing, depending how many people it takes out of the little system…. If you take people off the floor …, there are people not being looked after elsewhere **(NH B; ID15)**

*Sub-theme 4.4: Expectations of quality of MXS.* Participants were concerned about the safety of the procedure in the NH and whether the quality of MXS equalled that conducted in hospital or community radiology.

People may be a little bit suss about the fact is there may be radiation involved. … when they’re using those big machines, everybody’s behind glass et cetera, and you’re going, well, ‘what sort of protection is there when you’re using the mobile X-ray?’ **(NH B; ID14)**

Is it the same state-of-the-art equipment, … as if you were going into the [hospital]? So as long as it is doing the same thing, … that would be fine **(NH B; ID35)**

They also highlighted the need for radiology staff to be trained and skilled in communicating and managing residents within the NH environment.

There are some people who just have no idea how to be with someone who is actually even old, because … they’re uncomfortable with older people. People with dementia, you have to realise that it’s probably going to take more time than in doing your job and whizzing out again. So the staff would have to be very well trained to be that sort of person that could be with old people **(NH B; ID15)**

*Sub-theme 4.5: Expectations about MXS availability.* Participants expressed concerns about the timely availability of MXS in urgent situations. They were more willing to wait for less urgent X-rays (providing it was clinically safe). Participants emphasised that the quality of treatment should not be compromised. They highlighted that the general practitioner would influence their choice, if treatment was not necessary in hospital, pain was manageable and the MXS was available within the timeframe indicated by the general practitioner .

If it’s just a routine thing, that’s fine, and the patient or Mum’s not in pain, that’s fine ... She would be happy to just wait until they go there **…** if a resident’s in pain, … they should be followed through somewhere else, because they can’t wait four … or two hours **(NH A; ID31)**

If it’s not urgent, maybe a couple of weeks, like a normal medical appointment. … So it would be good if they could come … to urgent cases. … But I’d assume again the doctor would make the call, wouldn’t they? They’d just send them to the hospital if … if they knew no one [MXS] was scheduled to come **(NH B; ID35)**

But if it’s that critical, … I would expect the doctor to say it needs to be done today and if you [MXS] can’t do it, then she needs to go to A&E [accidental and emergency] or to a [community radiology] **(NH C; ID28)**

## Discussion

We have previously demonstrated that stakeholders consisting of health and aged care professionals, viewed MXS as a means for avoiding hospital transfers and reducing demand on hospital emergency departments [14]. Providing further evidence that such services will likely become an integral part of the health-system, a substantial finding from this study was that the use of MXS within NHs had strong appeal for ICs as a means of managing residents’ healthcare-in-place in the NH ICs, like residents, valued residents avoiding transfer for healthcare and remaining in the supportive NH environment [15]. This potentially reduced ICs’ physical and emotional burden; residents echoed these views [15]. ICs also recognised that MXS could reduce demand on the acute care health system burden, but the call-out fee for the MXS could result in health inequities. ICs suggested that NHs and MXSs needed to consider ensuring delivery of a successful MXS.

The use of MXS had strong appeal for ICs. ICs, as did residents [15], valued the environment that the NH provided; routines, activities, familiarity, comfort and security, which, in turn, fostered resident well-being. Therefore, ICs preferred residents remaining in the environment that optimised their well-being, without disruption, by receiving healthcare-in-place in the NH. Our findings are supported by residents in our study [15] and Kjelle & Lysdahl’s systematic review [21]. Further, NH resources could make it more comfortable for residents to have radiology in a NH than in fixed radiology sites. We are not aware that this has previously been documented.

Many ICs are heavily invested in the well-being of residents. A recent American study reported more than half of NH residents received help from ICs for self-care and mobility needs [22]. In fact, the need to help with self-care was higher for residents compared to community-dwelling older people [22]. Identifying methods to reduce carer burden should be a priority goal of any health or aged care service model. ICs in this study reported a potential for reduced IC burden with MXS, as they would not have to accompany residents to hospitals. When residents are transferred to hospital, ICs are called upon to take increased responsibility for residents’ needs. Indeed, residents and the health system rely on ICs to provide escort and transport, orientate, care, advocate and communicate for the resident [2, 19, 23]. ICs willingly provide this support because of their priority for residents’ well-being and because of familial obligation [24]. However, as our data demonstrate, the experience can be demanding and stressful (such as responding to distressed loved ones), disrupting routine and sleep,, and coping with waiting and uncertainty [22]. Thus there can be potential exacerbation of caregiver burden for some ICs[25]. Further, ICs who are still working need flexibility in work and other commitments, and, though participants did not report this, the potential for lost earnings (especially children of residents) and economic productivity is a reality [18]. Research with residents demonstrate that when they are unwell, they also prefer that their ICs are not additionally burdened [15]. The literature does not explicitly acknowledge the many costs to ICs from resident transfers and so the benefits of MXS are not fully identified. For example, a study investigating resident transfer cost to emergency department included transport costs but not informal carer cost (financial or non-financial) [26]. Future studies investigating the benefits of hospital avoidance strategies and the provision of healthcare in NH should take into consideration the impact on IC burden.

Norwegian data reported a significantly reduced public health cost associated with MXS use in aged care (mostly from reduced transportation and hospitalisation) [27]. However, this study evaluated state funded MXS and did not consider costs for users. One study of a MXS, operated out of a metropolitan Australian hospital, demonstrated a 11.5% reduction in emergency department presentations from NH over 12 months (using a before-after retrospective design). This implies decreased costs for the acute care sector [10]. Stakeholders in our study considered that MXS in NH would reduce costs for the acute care sector [14]. However, studies have not considered user’s cost considerations [10, 27]. Those cost considerations have been highlighted in this study. Many ICs in this study were willing to pay for a call-out fee, so that residents could access a MXS and avoid transfer to hospitals or radiology facilities. However, they highlighted that the call-out fee would be a barrier for some residents unable to meet these costs, risking health inequities; sentiments also voiced by residents [15]. The Australian Government rebate to subsidize the MXS call-out fee for limited indications (since November 2019) goes some way towards redressing this potential inequity in access to healthcare. However, if MXS providers continue to charge out-of-pocket gap payments, which may be the case for some providers, if for instance the rebate does not meet the cost of transportation, it still needs to be met by residents or ICs. Hence, cost could remain a barrier for some economically vulnerable residents [11]. It is therefore important that trends in out-of-pocket costs to consumers continue to be monitored with timely government intervention to address issues of inequity.

While voicing their strong support for MXS being provided in NHs, ICs expressed concerns and expectations about aspects of the service that would need to be addressed for successful implementation. Many ICs, like residents [15], had low-level awareness of MXS attending NH residents on-site. It is a human right of aged care consumers (or their ICs) to know of such services [28], in order to make informed choices. ICs also thought that NH staff should be knowledgeable about how the MXS operates, including booking and payment processes. NH should keep a record of willingness to pay an upfront fee, as NH do in documenting other preferences; this is the only publication documenting this. ICs noted, as did residents and stakeholders, that NH staffing capacity could be a major challenge to the widespread uptake of MXS [14, 15]. NH staff already work with many competing demands placed on them and, currently, many Australian NH are not staffed with sufficient registered nurses [29]. The MXS process, whilst reducing burden on ICs, shifts the care burden to NH staff. For example, ICs assume that NH staff could provide reassurance for residents during the imaging process, at the same time noting this may take staff away from caring for other residents. Where healthcare is delivered in NH, there could be shift of the burden from acute care hospitals (with many registered nurses, doctors and allied health) to NH organisations without the compensatory increase in staffing allocation to support this healthcare provision [14]. With our previous research, stakeholders noted that beyond the imaging process, if residents require monitoring, or resident’s needs escalate, this may exceed NH staff capacity [14]. Therefore, while MXS could support the delivery of healthcare-in-place and have a role in hospital avoidance, safe and quality delivery of healthcare is highly reliant on NH staff capacity and skills [29]. Hospital avoidance or substitution services could consider additional short-term resourcing to NH, taking into account the acuity and increased health and care needs of residents, to boost staffing to enable the delivery of healthcare-in-place with support consultation from hospital services. Further, information should address ICs’ concerns of quality and safety (such as radiology imaging and exposure to radiation) and expectations regarding the skill set of MXS radiographers working effectively with residents, as noted and observed by residents and radiographers [15, 30, 31]. Other concerns were ensuring that the availability and healthcare received by the resident was not compromised or delayed by the choice to receive the diagnostics and care in the NH rather than being transferred to hospital for investigation and management. These are consistent with the perceptions and observations of residents [15, 30]. ICs would also like the booking time to be confirmed with them. Models of care shifting healthcare away from hospitals to NH ought to be progressed with this consideration front of mind, given its importance to both residents and ICs.

A major strength of this study is the converging findings from ICs, residents and stakeholders [14]. Despite our preference, only one interview was conducted with an IC of a resident who had received a recent MXS, and so this knowledge remains a gap. The findings therefore may not apply to perspectives of ICs of residents who have experienced a MXS. A study is underway exploring the understanding of ICs whose residents have experienced MXS and the likelihood of identifying such participants are higher given that the Medicare Benefit Schedule rebate has been in place for more than two years. The majority (i.e. 80%) of participants were adult children of residents, who might have different perspectives to spouses and further research with ICs who are spouses will also be important.

## Conclusions

Our findings suggest there is optimism from ICs that MXS in NHs can improve the healthcare experience of residents, without unnecessary transfer to external X-ray services and additionally, can reduce IC burden. However, further engagement is needed to understand the concerns of ICs of residents who have experienced MXS. Involving ICs in the co-design of services will aid the successful implementation of MXS in NH and, similarly, there is a need also to hear from ICs as healthcare programs to support healthcare in NH are progressed. From a policy perspective, there is a need to monitor utilisation trends and resident’s out-of-pocket costs not covered by the Medicare Benefit Schedule rebates in the longer term to ensure that access to the service is equitable for all residents.

## Electronic supplementary material

Below is the link to the electronic supplementary material.

Supplementary Material 1

Supplementary Material 2

## Data Availability

Requests for data should be directed to the corresponding author (joanne.dollard@adelaide.edu.au) and ensuing research will require collaboration with the chief investigators (RV and JD). Any requests will be assessed for scientific rigor by this investigator team. A request for ethics approval and/or amendment must be prepared by the requestor in line with the requirements of Human Research Ethics Committee of the University of Adelaide. This ethics amendment must be approved by the Human Research Ethics Committee of the University of Adelaide. A data sharing agreement will need to be put in place. The requestor will be responsible for providing the necessary funding required for this process, including for the provision of data. Given that the grant funding for the project is in place to the 31st of December 2022 and there may be analyses continuing, then data sharing is embargoed till the 30th of March 2023.

## Abbreviations

MXS: Mobile X-ray service
NH: Nursing Home
ED: Emergency Department
CR: Community Radiology
IC: Informal carers
