# Supplementary material for: Mobile X-ray services in nursing homes as an enabler to healthcare-in-place for residents: informal carers’ views
Source: BMC Geriatr. 2023 Jul 25;23:458. doi: 10.1186/s12877-023-04130-7 (PMC10369836; doi:10.1186/s12877-023-04130-7)
Supplement: Supplementary file 1 — Supplementary Material 1 [file 12877_2023_4130_MOESM1_ESM.docx]

**Supplementary Material 1**

**Informal carer interview guide**

Research objectives: To explore with residents and family member:

1. What was important to their lives
2. Their knowledge of what mobile radiology services to RACF are, what they believe the role of such a service is as well as the value of such a service in relation to what was important to their lives
3. Their perceived benefits (including value), costs, risks, barriers and facilitators and for those who have accessed mobile x-ray their experiences of mobile x-ray services
4. Their perspective as to what the role of such a service should be (including its use for hospital avoidance and if this changes when there is ramping or with the pandemic), how important it is to them and what factors (including safety factors) ought to be considered in relation to the delivery of mobile radiology to residential aged care residents.
5. Their willingness to pay for a mobile x-ray

|  | Core questions | Major prompts |
| --- | --- | --- |
| 1 | First, could you please tell me a little about [name of family member]    Could you tell me what you consider is important to their life now? |  |
| 2 | What do you know about mobile x-ray service?    In what ways (or how) would mobile x-rays be useful where [name of family member] lives?    Thinking about what you have said that is important to [name of family member’s] life, how might mobile x-ray service help them to maintain those things? | (if they don’t know, then give a brief explanation) |
| 3 | (for those who have experienced mobile x-ray) Can you tell me about [name of family member’s] experience in having a mobile x-ray? Can you walk me through this?  What were the pros and cons (actual or potential)    (for those who have not experienced mobile x-ray) What do you think about mobile x-ray services being offered to residents like [name of family member] living in an aged care home?  What would be the pros and cons for residents to have a mobile x-ray? | What are the   - Benefits and value of having mobile x-ray - Costs or burdens about having mobile x-ray - Risks or concerns about having mobile x-ray - Barriers to receiving mobile x-ray - Facilitators to receiving mobile xray |
| 4 | What health conditions or situations would you prefer [name of family member] to have a mobile x-ray?    What health conditions or situations would you prefer [name of family member] to go to ED for an X-ray?    What circumstances would you want to avoid [name of family member] going to ED?  How important is having a mobile x-ray to you and [name of family member]?    In providing a mobile x-ray service to residents in aged care homes, what factors need to be considered?    In having a mobile x-ray, what worries or concerns might you have for [name of family member]? | - Use for hospital avoidance and if this changes when there is ramping or with pandemic - Intrinsic reasons (dementia, mobility, sensory loss, frailty, weakness) and urgency of condition - Extrinsic reasons – ramping, pandemic - Time of day and day of week        - Family considerations - RACF staff being able to manage situation (workload, skills) Waiting times for GP to attend Waiting times for having mobile x-ray and results being provided to GP Waiting times for treatment |
| 5 | Would you be willing to pay for mobile x-ray services so that [name of family member] can have their x-ray in this aged care home?  Would your response change if it was an emergency situation?  If yes, how much would you be willing to pay?f |  |
|  | If there anything else you would like to add? |  |
