# Supplementary material for: Mobile X-ray services in nursing homes as an enabler to healthcare-in-place for residents: informal carers’ views
Source: BMC Geriatr. 2023 Jul 25;23:458. doi: 10.1186/s12877-023-04130-7 (PMC10369836; doi:10.1186/s12877-023-04130-7)
Supplement: Supplementary file 2 — Supplementary Material 2 [file 12877_2023_4130_MOESM2_ESM.docx]

**Additional File 2.** Themes, sub-themes and supporting quotes.

| Themes;  Sub-themes | Example quotes |
| --- | --- |
| Theme 1: ICs’ priority of maintaining resident well-being could be facilitated by using MXS | |
| Sub-theme 1.1: Resident well-being was a priority to ICs | Dad requires familiarity … the need to be safe. He needs to be around people who enforce a routine for him. He is better when his day staff are on. He copes better **(NH A; ID05)**  Yes, but he just seems to think, ‘oh, it's all right here’. He can walk around or sit where he wants and walk outside the little garden, have a look if he wants to, and not be restricted as far as his movement is concerned (**NH B; ID13**)  She's really enjoying some of the activities that they provide now. So that's made her more sociable, she's meeting new friends **(NH C; ID37)**  She knows that she's safe, that she has help when she needs it and that there's people - company around her **(NH C; ID27)**  She's comfortable and secure in her current environment …, it's a major exercise and a very painful one inserting her in and out of a car **(NH C; ID23)** |
| Sub-theme 1.2: Perceived benefits of mobile X-ray service to residents’ well-being | They don’t have to be taken anywhere out of their comfort zone, to be in a situation where it’s unfamiliar, especially with people with dementia **(NH B; ID15)**  That would probably save her the stress of being loaded in - get an ambulance there, wait for it, load her, come down, wait around a waiting room, come back **(NH A; ID09)** |
| Sub-theme 1.3: Using equipment available in the NH to manage MXS for resident benefit. | If they needed a lifter or anything like that, they would have it there. … when you go to the hospital they don't always have that equipment **(NH B; ID35)** |
| Theme 2: Reduced carer burden | |
|  | I have to be the person that speaks for him, because mostly no one understands him. That’s distressing in itself **(NH A; ID04)** |
|  | Well, it’s waiting with her because she’s not reliable to stay by herself. If I walked away and left her and I say ‘just sit there’, she’s just liable to walk off, because she can’t see me now, so she just goes (**NH C; ID28)** |
|  | I think if she’s in her own zone, and with people that she knows, I wouldn’t necessarily have to be there **(NH D; ID19)**  Just as support for him, but it also gives me the opportunity to speak to the X-ray person as well and know what they're looking for. Then I've got the contact straight away with the staff as well, so make sure everything's in train, ready **(NH C; ID36)** |
| Theme 3: ICs economic considerations of using MXS | |
| Sub-theme 3.1: Cost benefit to health system | If it's going to help our hospitals to decrease the number of people being sent there **(NH C; ID27)** |
| Sub-theme 3.2: Cost benefits of call-out fee for mobile X-ray service to individuals | She’s … a government pensioner, so I imagine it would be fully subsidised **(NH C; ID23)**  I reckon if it got over $150, I would think that that’s getting to be a bit much. …I would pay anything - or Mum could pay anything in preference to having to go to the hospital **(NH D; ID18)**  I’d say up to $500. Something like that. If it was going to be much more comfortable for her (**NH D; ID20**)  If it’s an X-ray that the doctor says is vital and urgent and she’s needs it now, then I’d probably pay it without too much drama, but if the doctor is umming and erring and saying, ‘oh I think she might need an X-ray’ …, then I would say, ‘well, no we’ll go to hospital or we’ll wait’ **(NH D; ID21)** |
| Theme 4: Pathways to translating MXS into NH to meet residents’ and ICs’ needs and expectations | |
| Sub-theme 4.1: Awareness of and need for promotion of mobile X-ray service | All I knew is there were X-rays in hospitals, and that's about all I knew **(NH B; ID13)**  It was gossip floating right through the entire complex, that this elderly gentleman had had a mobile X-ray and she [resident] was quite amazed that such a thing existed. She asked me if I'd heard of it and I said ‘no, I hadn’t’. She said ‘I think it's a marvellous idea, but apparently it didn't work, so he still had to be transported somewhere else to have the X-ray.’ **(NH C; ID37)** |
| Sub-theme 4.2: Effective processes to using MXS | There'd have to be induction on how a program like that would work, of course **(NH A; ID05)**  We weren’t told how long, so we went ‘oh well, we’ll go home’ because we’d been there three or four hours already and we went home and kept ringing the home to find out whether or not they’d been, and they hadn’t. …That’s when the home chased them up then and found out that they’re not coming basically **(NH D; ID21)**  We rang their [MXS] phone and no-one would get back to us. … must have been busy or something and it might have been a Saturday **(NH C; ID23)** |
| Sub-theme 4.3: NH staff levels to manage MXS and care process | They do appear to have reasonable staff [numbers]. It’s always hard at busy times. There’s always times when whatever staff you have is never enough. I don’t think that would be a major problem in the [NH] **(NH C; ID28)**  There are a lot of residents who are also highly stressed. There are some who … get quite angry and aggressive. So, they’re [staff] stretched to the limit to cope with people there. So, for them, they may not be so keen on having to take out extra time to sit with somebody through the X-ray **(NH D; ID18)**  Based on Mum's experience with the staff, lack of staff …, but I would assume the nurse would have to be there and there's only one nurse for the entire complex each shift. Getting her to the patient's room at the time that the machine arrives, I think it might be a bit of a logistical nightmare for them, to tell you the truth **(NH C; ID37)** |
| Sub-theme 4.4: Expectations of quality of service | There's obviously the moderate danger from the X-rays as well that they have to take into account. …obviously - I think an over-exposure to X-rays is probably not good for long-term health and I imagine that's probably why you see the X-ray technicians go and hide behind the screen **(NH C; ID23)**  They definitely need people skills. If they're entering into someone's home, the room is their home and they're in pain or they're distressed, the radiographer needs to have the right people skills to handle that situation **(NH C; ID37)** |
| Sub-theme 4.5: Expectations about availability | Because people don’t fall over and break limbs on the Tuesday afternoon at three o’clock. The service has got to be available 24/7 **(NH D; ID21)**  It depends on if you're going to have a mobile unit that would cover maybe north, south and west, so that you've got one that doesn't go out of that - like their area. … that would help a bit because that would cut down waiting time **(NH C; ID27)** |

NH: Nursing Home
